# Effect of olive and date palm by-products on rumen methanogenic community in Barki sheep

**DOI:** 10.3934/microbiol.2022003

**Published:** 2022-01-27

**Authors:** Alaa Emara Rabee, Khalid Z. Kewan, Hassan M. El Shaer, Mebarek Lamara, Ebrahim A. Sabra

**Affiliations:** 1 Animal and Poultry Nutrition Department, Desert Research Center, Cairo, Egypt; 2 Genetic Engineering and Biotechnology Research Institute, University of Sadat City, Sadate City, Menoufia, Egypt; 3 Forest Research Institute, University of Quebec in Abitibi-Temiscamingue, Rouyn-Noranda, Canada

**Keywords:** sheep rumen, methanogenic archaea, illumina Mi Seq, olive cake, discarded dates, date palm fronds

## Abstract

Rumen methanogens prevent the accumulation of fermentation gases in the rumen and generate methane that increases global warming and represents a loss in animals' gross energy. Non-traditional feed resources such as the by-products of date palm (*Phoenix dactylifera*) and olive (*Olea europaea*) trees have received attention to be used in animal feeding. This study evaluated the impact of non-traditional feed resources including olive cake (OC), discarded dates (DD), and date palm frond (DPF) in sheep diet on rumen fermentation, diversity and relative abundance of rumen methanogens. Nine adult rams were assigned to three equal groups and fed three diets: traditional concentrates mixture (S1); non-traditional concentrate mixture (S2) based on DD and OC; and (S3) composed of the same S2 concentrate supplemented with DPF as a roughage part. The results showed that rumen pH was higher with S3 diet than the other two diets. However, the S1 diet showed the highest values of total volatile fatty acids (TVFA) and rumen ammonia. In addition, the proportions of acetic and butyric acids were increased, whereas propionic acid declined in S2 and S3 compared to the S1 diet. Rumen methanogens were dominated by *Methanobrevibacter* that showed a numeric decline by including DD, OC, and DPF in the animal diets. Principal component analysis (PCA) based on rumen fermentation parameters and relative abundances of methanogens genera showed three distinct clusters. Also, positive and negative correlations were revealed between methanogens genera and rumen metabolites. This study expands the knowledge regarding the effect of agricultural byproducts on rumen fermentation and the methanogenic community.

## 1. Introduction

Rumen fermentation relies on complex microbial groups, including bacteria, protozoa, fungi, and archaea, that work synergistically to convert ingested feed to volatile fatty acids, ammonia (NH_3_-N), hydrogen (H_2_), and carbon dioxide (CO_2_) [1]. Among rumen microbial groups, methanogenic archaea represent a small proportion of rumen microbial communities. They utilize formate as an energy source and use hydrogen to reduce CO_2_ to methane [2]–[4]. Removing hydrogen from the rumen environment by methanogens helps to activate microbial fermentation [5]. Methane production represents a loss in animal's gross energy; besides, it contributes to global warming [6]. Therefore, limiting the activity of the methanogenic community in the rumen could enhance the animals' feed efficiency and reduce environmental pollution [2].

Diet composition modulates the fermentation patterns and rumen pH; consequently, the structure of the methanogenic populations could be altered [4]. Therefore, understanding the changes in the rumen methanogens due to changing the diet type could lead to various strategies to improve animal efficiency and mitigate methane emission [7]. Next-generation sequencing (NGS) technologies are powerful techniques to study the composition and dynamics of rumen microbiota due to changes in the feeding system or by adding specific chemical inhibitors [8].

Barki sheep breed is a crucial part of food security in arid countries due to its adaptability to desert harsh conditions; however, it lacks the appropriate feeding systems to increase its productive performance [9]. Therefore, various combinations of locally available feed resources could be used to enhance animal productivity at a low cost [10].

The availability of local agricultural byproducts generated the primary interest to incorporate them in animal feed to fill the gap in animal feeding and avoid the environmental consequences [7],[9]. Large quantities of agricultural wastes such as OC, DD, and DPF are produced in arid countries; thus, these byproducts could replace the traditional feed resources [9]. Derivatives provided from date palm trees and OC are rich in phenolic compounds that influence the rumen microbiota and enzymes negatively besides their inhibitory effect on methane production [7],[9].

Several studies investigated the effect of OC, DD, and DPF individually on animal efficiency and rumen fermentation [11]–[13]. Besides, the methanogenic archaea were studied extensively in the rumen of different ruminant animals under different feeding systems [4],[5],[7]. However, the effect of non-traditional feed mixtures containing combinations of OC, DD, and DPF on the density and composition of methanogens in Barki sheep has not been studied yet. Therefore, the aim of this study was to (1): investigate the effect of the inclusion of OC, DD, and DPF in the diet of Barki sheep on rumen fermentation, diversity and structure of rumen methanogens, (2): explore the correlation between rumen fermentation parameters, enzymes' activities, and rumen methanogems.

## 2. Material and methods

### 2.1. Experimental diets

The experimental diets and their chemical compositions were previously described in Rabee et al. [9] and are included in supplementary Tables S1 and S2. Three dietary treatments were used in this study as follows: S1, a common concentrate mixture served as a control ration; S2, common non-traditional concentrate mixture including 10% olive cake (OC) and 60% discarded date palm (DD); and S3, the same concentrate mixture in S2 supplemented with roughage as ground date palm fronds (DPF) enriched with 15% molasses.

### 2.2. Rumen fermentation

The experiment was conducted at Maryout Research Station, Desert Research center, Alexandria, Egypt. In this experiment, nine adult Barki rams (3 years old with an average body weight of 42.3 ± 3 kg) were assigned into three treatments (n = 3) to determine the effect of experimental diets on rumen fermentation parameters, enzymes activities (cellulase and xylanase); and the relative abundance and diversity of rumen methanogens. Animals were adapted for individual feeding on the experimental diets for 15 days. Rumen samples were collected at 3h post-feeding using oral stomach tubing. Rumen pH was recorded using pH meter (WPA CD70). Then, rumen samples were frozen till further analyses. The study was approved and all samples were obtained according to the guidelines of Institutional Animal Care and Use Committee, Faculty of Veterinary Medicine, University of Sadat City, Egypt (Approval reference number: VUSC00008).

### 2.3. Gas and methane production

The experimental diets were evaluated for gas and methane production according to the method of Yusuf et al. [14] and Fievez et al. [15]. An exact 200 mg of each dry finely ground diet was placed into 100 mL glass bottles containing 20 mL of the medium (pH 6.8) and 10 mL rumen liquor that had been filtered through 4 layers of cheesecloth. The rumen liquor was collected from three ruminally-cannulated rams one hour before the morning feeding. Six bottles were set up for each treatment. The total gas and methane volume were recorded after 24 h of incubation and the values were corrected for the blank value and the gas yield values are expressed in mL per 200 mg of DM. After recording the ﬁnal gas volume at 24 h of incubation, 4 mL NaOH (10 M) was injected into each bottle to measure methane volume.

### 2.4. Rumen liquor analysis

Rumen ammonia and total volatile fatty acids (TVFA) were measured using Kjeldahl distillation [16],[17]. Furthermore, individual volatile fatty acids concentrations were assessed using a high-performance liquid chromatograph [18]. Cellulase and xylanase enzymes were determined by measuring the released reducing sugars using 3, 5-dinitrosalicylic acid [19],[20]. The unite of xylanase was defined as the amount of enzyme that releases 1 µmol of xylose per minute per mL. The unite of cellulase was defined as the amount of enzyme that hydrolyze filter paper to release 1 µmol of glucose per minute per mL.

### 2.5. DNA Extraction, PCR amplification, and sequencing

DNA extraction from rumen samples was conducted using i-genomic Stool DNA Extraction Mini Kit (iNtRON Biotechnology, Inc.) as described by Rabee et al. [9] and DNA was eluted in 50 µL buffer. The quality and quantity of extracted DNA were checked using gel electrophoresis and Nanodrop spectrophotometer. Subsequently, the archaeal 16S rDNA gene was amplified using primers Ar915aF (5-AGGAATTGGCGGGGGAGCAC-3) and Ar1386R (5-GCGGTGTGTGCAAGGAGC-3) [5]. The PCR amplification was carried out under the following conditions: 95 °C for 5 min; 30 cycles 95 °C for 20 s, 55 °C for 15 s, 72 °C for 5 min, and 72 °C for 10 min. The PCR-amplicons were purified and prepared for Illumina MiSeq sequencing according to the protocol of Comeau et al. [21] in Integrated Microbiome Resource (Dalhousie University, Canada).

### 2.6. Real-time quantitative PCR (qPCR)

Quantitative PCR (qPCR) was carried out to measure the total copy number of archaeal 16S rDNA in 1 µL of isolated DNA. Standards were generated using dilutions of purified DNA from *Methanobrevibacter ruminantium*, and *Methanosphaera stadtmanae* purchased from Deutsche Sammlung von Mikroorganismen und Zellkulturen (DSMZ), Braunschweig, Germany. The standard curve was generated using dilution series of the standards ranging from 10^1^ to 10^6^ copies of the 16S rDNA. The qPCR was conducted using Applied Biosystems StepOne system (Applied Biosystems, Foster City, USA). The archaeal specific primers Arch 1174-1195 F (5-GAGGAAGGAGTGGACGACGGTA-3) and Arch 1406-1389 R (5-ACGGGCGGTGTGTGCAAG-3) [5] were used to amplify DNA samples and diluted standards. The 10-µL qPCR reaction contained 1µL genomic DNA, 1 µL of each primer, and 7 µL of SYBER Green qPCR- master mix (iNtRON Biotechnology, Inc.). The qPCR conditions were 40 cycles of 95 °C for 15 s, and 60 °C for 60 s. The total copy number of archaeal 16S rDNA per 1 µL of DNA was determined relying on the linear relationship between the threshold amplification (Ct) and the logarithm of 16S rDNA copy numbers of the standards.

### 2.7. Bioinformatics analysis

Illumina paired-end raw sequence reads were processed in R (version 3.5.2) using the DADA2 pipeline as described by Callahan et al. [22]. Quality checks were conducted then clean reads were denoised, dereplicated, and filtered for chimeras to generate Amplicon Sequence Variants (ASVs). The taxonomic assignment of sequence variants was performed using a combination of the functions assign Taxonomy and assign Species and was compared using the SILVA reference database.

### 2.8. Statistical analysis

The differences in the diversity and relative abundance of rumen archaeal genera as well as rumen fermentation parameters and enzymes due to changing the diet type were assessed using one-way ANOVA and the differences were statistical at P < 0.05. A post hoc Duncan test was carried out to determine the significant differences. Principal component analysis (PCA) was conducted based on the relative abundance of rumen methanogens and rumen fermentation parameters to compare the clustering pattern of rumen samples. Pearson correlation analysis was used to identify the correlation between methanogens genera and rumen fermentation parameters and the correlation scores were visualized as a heatmap. The statistical analyses were conducted using the SPSS v. 20.0 software package [23] and PAST [24]. The raw sequence reads were deposited to the sequence read archive (SRA) under the accession number: PRJNA745087.

**Table 1.** Rumen fermentation parameters, rumen enzymes activities, and methanogens population (Log10 copies/µL 16S rDNA) in the rumen of sheep fed different diets (Mean ± SE).

Item	S1		S2		S3		Overall Mean	SEM	P value
	Mean	SE	Mean	SE	Mean	SE			
Rumen fermentation parameters									
pH	5^a^	0.05	5.2^a^	0.04	5.91^b^	0.12	5.35	0.14	<0.0001
NH3N, mg/dL	6.6^b^	0.04	3^a^	0.05	3^a^	0.06	4.2	0.6	<0.0001
TVFA, meq/dL	13.1^b^	0.4	8.8^a^	0.24	8.75^a^	0.08	10.2	0.73	<0.0001
Acetic%	51.3^a^	0.3	54.3^a^	1.2	66.7^b^	2.3	57.4	2.5	0.001
Propionic%	41^c^	0	35^b^	2.5	20^a^	0.6	32	3.2	<0.0001
Butyric%	7.7	0.3	10.7	1.8	13.3	1.7	10.5	1.09	0.086
A/P ratio	1.25		1.55		3.3				

Enzymes activity, IU/mL									
Cellulase	7.7	1.3	10.5	0.86	9	0	9.06	0.6	0.168
xylanase	6.5	1.75	6	2.8	3.2	2.2	5.2	1.25	0.567

Archaeal copy number; Log 10 copies/µL 16S rDNA									
Log10	5.8	0.148	5.2	0.6	6.2	0.6	5.7	0.28	0.415

*Note: A: Acetic; P: Propionic.

## 3. Results

### 3.1. Rumen fermentation indicators

Dry matter feed intake expressed as g/kg^0.75^ (kilogram metabolic body weight) was 61.9 for S1 group, 66 for S2 group, and 62.7 for S3 group. The effect of diet type on rumen pH, TVFA, and ammonia is shown in Table 1 and Supplementary Table S3. Diets S2 and S3 showed higher pH than S1 diet. However, the diet S1 showed the highest values of TVFA compared to the other diets. Additionally, TVFA concentration was found to be inversely correlated with the rumen pH. Moreover, non-traditional diets declined the rumen ammonia (mg/dL), wherever S1 group revealed the highest concentration compared to S2 and S3 groups. The sheep group that received the S3 diet showed a significantly higher concentration of acetic and butyric acids; meanwhile, the S1 group showed higher propionic acid percentage (Table 1). The total methanogens population (Log10 of 16S rDNA copy number/µL) was greater in the sheep group that received the S3 diet compared to the other groups (Table 1). Diet type had no significant effect on the activities of rumen enzymes (IU/mL) (Table 1). The greatest cellulase production was obtained in animals fed the S1 diet followed by S3 and S2, respectively. Regarding xylanase production, the sheep group fed the S1 diet showed a higher xylanase production than other groups.

**Table 2.** Gas yield and methane production of experimental diets under investigation.

Item	S1		S2		S3		Overall Mean	SEM	P value
	Mean	SE	Mean	SE	Mean	SE			
Total gas yield, mlL0.2 g DM	20	0.32	17	0.53	18.4	1.03	18.5	0.55	0.06
Methane, mL/0.2 g DM	4.05^a^	0.04	4.50^a^	0.2	6.75^b^	0.16	5.09	0.42	<0.0001
Methane/TG, %	20.27^a^	0.37	26.49^b^	0.35	36.73^c^	0.55	27.83	2.4	<0.0001
CH4 energy, MJ/d	1.91^a^	0.05	2.50^b^	0.05	2.83^c^	0.06	2.4	0.13	<0.0001
CH4 energy/GEI, %	10.32^a^	0.26	12.51^b^	0.28	16.03^c^	0.54	12.95	0.85	<0.0001

### 3.2. Total gas yield and methane production

The control diet (S1) showed a higher value of gas yield as compared to the other diets (Table 2). However, the vice versa was observed for methane yield as a percent of total gas yield and also for energy loss as a percent of gross energy intake.

**Figure 1.**
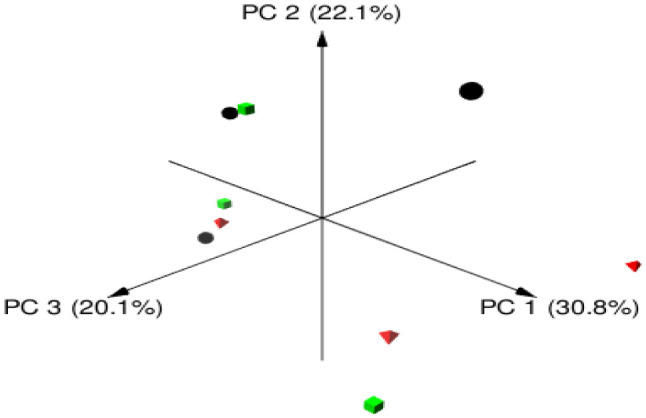
Principal coordinates analysis (PCoA) of archaeal community in the rumen of Barki sheep. PCoA was carried out between three sheep groups fed different diets: black circles for S1 group, red triangles for S2 group, and green squares for S3 group.

### 3.3. Diversity of rumen methanogens

Illumina sequencing of rumen methanogenic archaea in sheep generated 193885 high-quality sequence reads with an average of 21542 reads per sample. The mean of ASVs was similar for S1 and S3 (65) and was different (65 and 46) between the S2 and S3 groups without significant difference (Table 3). Moreover, different alpha diversity indexes, including Chao, Invsimpon, and Shannon, were used to determine the diversity of rumen methanogens in this study. Sheep in groups S1 and S3 showed similar and higher Chao and Shannon values compared to the S2 group. At the same time, the S3 group showed the highest Invsimpon value without significant difference. Beta diversity of rumen methanogens was calculated and visualized using principal coordinate analysis based on the Bray-Curtis distances. No clear separation of rumen samples due to changing the diet was observed (Figure 1).

**Table 3.** Number of ASVs and alpha diversity indices of methanogenic community in the rumen of sheep under different diets (Mean ± SE).

Index	S1		S2		S3		Overall Mean	SEM	P value
	Mean	SE	Mean	SE	Mean	SE			
AVSs	65	10.7	46	7.8	65	14.3	58.5	6.4	0.461
Chao1	65	10.5	46	7.8	65	14.3	58.7	6.4	0.453
Shannon	2.5	0.2	2.07	0.4	2.5	0.56	2.4	0.2	0.650
InvSimpson	8	1.7	5.3	2.1	8.9	4.4	7.4	1.6	0.687

### 3.4. Structure of methanogenic community

**Figure 2.**
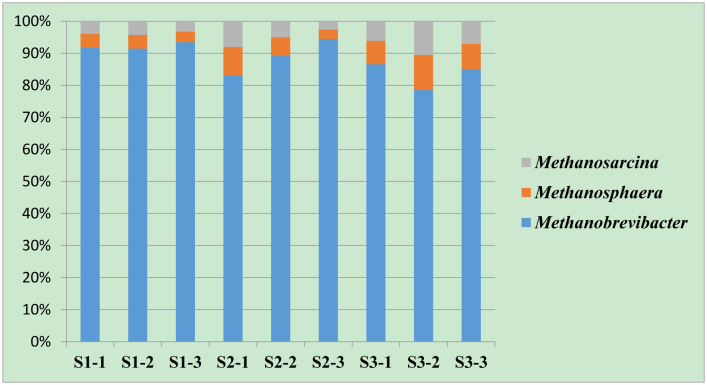
The relative abundances of methanogenic genera in the rumen of three sheep groups fed different diets (S1, S2 and S3).

The methanogenic community in the sheep rumen was assigned to phylum Euryarchaeota, which was classified into two orders, Methanobacteriales and Methanosarcinales. All the reads under order Methanobacteriales were classified to family Methanobacteriacea that was assigned to two genera, *Methanobrevibacter* and *Methanosphaera* (Table 4). *Methanobrevibacter* was the most predominant genera in all groups (Table 4, Figure 2). Group S1 revealed the highest representation of *Methanobrevibacter*, followed by S2 and S3, respectively. In contrast, genus *Methanosphaera* was increased in the relative abundance when OC, DD, DPF replaced the traditional feed mixture. Group S3 revealed the highest representation of *Methanosphaera* followed by S2 and S1, respectively. Sequence reads assigned to order Methanosarcinales were classified to family Methanosarcinaceae and genus *Methanosarcina* that was higher in sheep group S3, followed by S2 and S1, respectively.

**Table 4.** The relative abundance (%) of methanogenic genera in the rumen of sheep fed different diets (Mean ± SE).

Genera	S1		S2		S3		Overall Mean	SEM	P value
	Mean	SE	Mean	SE	Mean	SE			
*Methanobrevibacter (%)*	92.2	0.62	89	3.26	83.4	2.44	88.2	1.7	0.096
*Methanosphaera (%)*	4	0.32	5.8	1.7	8.7	1.1	6.2	0.9	0.075
*Methanosarcina (%)*	3.8	0.3	5.2	1.56	7.9	1.3	5.6	0.8	0.127

### 3.5. Effect of diet type on rumen fermentation and relative abundance of rumen methanogens

**Figure 3.**
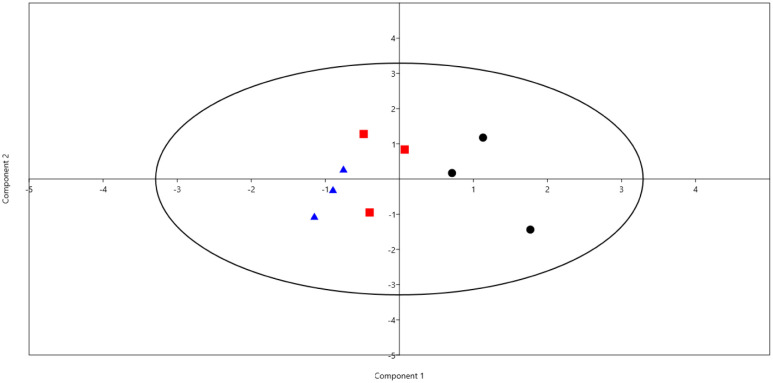
Principal component analysis (PCA) based on the relative abundances of rumen methanogens and rumen fermentation parameters in the rumen of three sheep groups fed different diets; black circles S1 group, red squares for S2 group, and blue triangle S3 group.

Principal component analysis (PCA) based on the relative abundance of rumen methanogens and rumen fermentation parameters separated the rumen samples into three distinct groups as shown in Figure 3. The relative abundance of *Methanobrevibacter* and the concentration of rumen ammonia, acetic acid, propionic acid, and xylanase enzymes were the most important parameters that drove the differences between animals.

### 3.6. Pearson correlations between rumen methanogens and rumen metabolites

**Figure 4.**
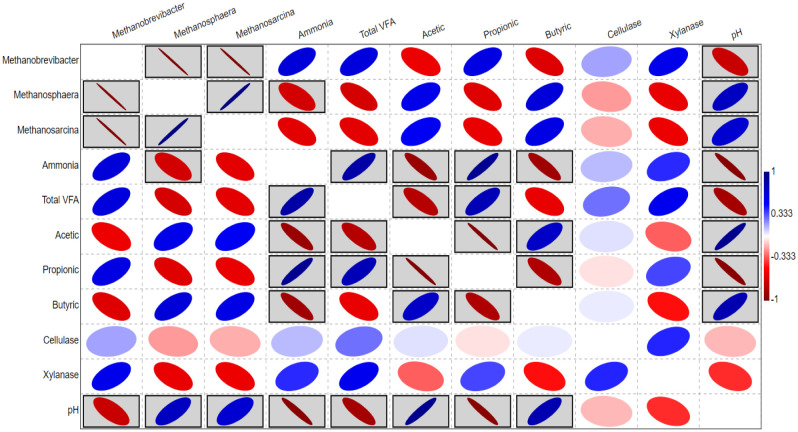
Heatmap based on Pearson correlation coefficients between the relative abundances of rumen methanogens genera, rumen fermentation parameters, and rumen enzymes. The black boxed ellipses refer to the significant correlations at P < 0.05.

Correlation analysis (Figure 4) showed some positive and negative correlation relationships between the relative abundance of methanogens genera and the rumen fermentation parameters. For example, *Methanobrevibacter* was positively correlated with rumen ammonia, TVFA, propionic acid, and xylanase, but negatively correlated with acetic acid, butyric acid, pH. Moreover, there was a positive correlation between the methanogens genera, *Methanosphaera* and *Methanosarcina*, and acetic acid, butyric acid, and pH. In addition, a negative correlation was observed between *Methanosphaera* and *Methanosarcina* and ammonia, TVFA, propionic acid, and xylanase.

## 4. Discussion

### 4.1. Experimental diets and feed intake

Rumen microbiota work symbiotically and form a complex network of metabolic pathways to facilitate the rumen fermentation of ingested feed [25]. Also, rumen microorganisms share similar growth requirements, and the end products of one group may be forming the growth substrates for another group [1]. Rumen methanogens always interact with hydrogen-producing or utilizing microorganisms [2], which are mainly influenced by diet composition [3]. In this study, the common feed mixture in group S1 was replaced partially by olive cake, and discarded dates; besides, date palm fronds were used as forage in group S3. Previous studies explained that the common concentrate feed mixture has low-fiber content and is rich in crude protein, energy, and soluble carbohydrates [4]. In the same time, olive cake and date palm fronds are described as low-quality feedstuffs with high contents of lignocelluloses and phenolic compounds and low contents of protein and energy [11],[13],[26],[27]. Furthermore, the discarded dates are considered an energy source due to high sugar and low crude protein content and it has a higher content of phenolic compounds [11],[28],[29].

### 4.2. Rumen fermentation

Higher rumen pH in group S2 and S3 was also indicated in previous studies that used olive cake and palm fronds in animals' diets [12],[13],[26]. The decline in rumen pH under optimum point affects rumen fermentation adversely, especially the cellulose degradation [30]; consequently, animal performance could be decreased. The concentrate feed mixture consists of rapidly fermentable carbohydrate that encourages the microbial activities and TVFA production that declines the rumen pH; in contrast, the higher dietary fiber has a neutralizing effect on rumen PH [31],[32]. This explanation supports the low pH, and higher TVFA production in the S1 group fed the common concentrate mixture (Table 1).

Acetic and butyric acids were increased, but propionic acid was decreased (Table 1), which agrees with previous studies [33],[34] that explained that high-fiber diets stimulate acetic production by encouraging fibrolytic bacteria; while, starchy diets stimulate propionic production. In addition, Khezri et al. [35] noticed that DD boosted the butyrate production. Higher butyric acid could be attributed to the presence of cellulolytic bacteria “*Butyrivibrio*” whose activity was stimulated by the high fiber in S3 [34]. The high protein content in the S1 diet was associated with the higher rumen ammonia (Table 1) that was declined by the inclusion of OC, DD, and DPF in the sheep diet [13],[36]. In addition, Rajabi et al. [37] reported that DD decreased the rumen ammonia and TVFA. The activities of xylanase and cellulase (Table 1) varied slightly by the inclusion of OC, DD, and DPF in the sheep diet. Higher cellulase and xylanase production could be attributed to the abundance of cellulolytic and xylanolytic bacteria [38],[39]. On the other hand, Kala et al. [40] illustrated that cellulase and xylanase in the buffalo rumen were not affected by diet type. Kamra et al. [41] showed that rumen enzymes follow the activities of microbial groups involved in rumen fermentation. These speculations support the results of rumen enzymes.

### 4.3. Gas yield and methane production

The high gas yield in S1 compared to S2 and S3 diets (Table 2) probably resulted from high soluble carbohydrates and the supply of N that support the growth of microorganisms [42],[43]. The main component affecting methane production is the type of carbohydrate and the relative rate of fermentation. The highest methane yield was observed in the high forage diet, S3 followed by S2 and then S1. These results are in agreement with Van Soest [44], who indicated that a high grain diet with soluble carbohydrate increase the passage rate and decline the rumen pH, which inhibits the rumen methanogens. The present result of loss of energy expressed was lower than that recorded for growing lambs (21.15%) fed berseem hay supplemented with concentrate feed mixture [45].

### 4.4. Population and structure of methanogens community

The higher methanogenic population in the S3 group is consistent with that found by Rabee et al. [4] on the high-forage diets. High-fiber diets stimulate fibrolytic bacteria such as *Fibrobacteres* that produce hydrogen and methyl groups for methanogenesis [46]. Starchy diets stimulate the genus *Prevotella* that utilize hydrogen and produce propionate that depresses methanogenesis in the rumen [4],[47], which might illustrate the lower methanogenic population in S1. García-Rodríguez et al. [13] found no variation in methanogens population after incorporation of OC in animal diet in In vitro study. Romero-Huelva et al, [48] indicated no relationship between the abundance of methanogens and methane emission in the rumen.

Diet type did not affect alpha diversity indeces, which agrees with previous study [4]. Additionally, the methanogenic community in the current study was classified into three genera (Table 4 and Figure 2), *Methanobrevibacter*, *Methanosphaera*, and *Methanosarcina* which is similar to results on goats and camels [4],[5]. Also, genus *Methanobrevibacter* was dominant in all the rumen samples, which is in agreement with previous studies on a wide range of ruminant animals [5],[49],[50]. The relative abundance of methanogenic genera in our study has experienced slight variations due to changing the diet type. This trend was also observed by Jeyanathan et al. [50], and Rabee et al. [4] and was supported by results of PCA (Figure 3) that showed that rumen samples were distinctly separated based on rumen fermentation parameters and relative abundance of methanogens genera.

Animal diet is the main driver of the changes in the rumen microbiota that ferment a wide range of substrates, including cellulose, hemicellulose, protein, and pectin [4]. Consequently, different gases, soluble sugars, and organic acids are generated including hydrogen, carbon dioxide, acetic, butyric, and glucose, which are required in methanogenesis [2]–[5]. Therefore, the variation in the density and relative abundance of methanogens in the current study could be attributed to the availability of the hydrogen or other substrates required for methanogenesis [5],[51]. These speculations illustrate the variations in the relative abundance of methanogens in the current study and are supported by positive correlations between the archaeal genera (*Methanosphaera* and *Methanosarcina*) and the acetic acid (Figure 4), which provides a methyl group for methanogenesis [52]. In the same time, both genera showed a negative correlation with propionic acid that is in the line with previous conclusions [53],[54]. The production of propionic in the rumen utilizes the hydrogen, the primary substrate for the methanogenesis, which influences the methanogens adversely [34].

The relative abundance of *Methanobrevibacter* was decreased numerically by raising the concentration of acetic acid. This finding could be interpreted by the presence of phenolic compounds in OC, DD, and DPF that affected relative abundance of *Methanobrevibacter* and methane production negatively [55]–[57], since genus *Methanobrevibacter* is active in methane production [52]. This finding highlights OC, DD, and DPF as promising feed resources to modulate methanogens community. The negative correlation between the archaeal genera (*Methanosphaera* and *Methanosarcina*) on one side and xylanase enzyme on other side could be explained by higher representation of xylanololytic bacteria such as *Prevotella* that utilizes hydrogen and affect methanogens negatively [53],[54].

## 5. Conclusion

Agricultural byproducts such as OC, DD, and DPF could be suitable alternatives of traditional feed resources and would be advantageous for the efficient use of available resources. Inclusion agricultural byproducts in animal diets affected the rumen fermentation, methanogenic community, and had an adverse effect on some rumen methanogens.
