# Supplementary material for: Effect of olive and date palm by-products on rumen methanogenic community in Barki sheep
Source: AIMS Microbiol. 2022 Jan 27;8(1):26–41. doi: 10.3934/microbiol.2022003 (PMC8995188; doi:10.3934/microbiol.2022003)
Supplement: Supplementary file 1 [file microbiol-08-01-003-s001.pdf]

AIMS Microbiology

AIMS Microbiology, 8(1): 26–41.
DOI: 10.3934/microbiol.2022003
Received date: 02 December 2021
Revised date: 09 January 2022
Accepted date: 24 January 2022
Published date: 27 January 2022

http://www.aimspress.com/journal/microbiology

Research article

Effect of olive and date palm by-products on rumen methanogenic community in Barki sheep

Alaa Emara Rabee[1,*], Khalid Z. Kewan[1], Hassan M. El Shaer[1], Mebarek Lamara[3] and Ebrahim A. Sabra[2]

[1] Animal and Poultry Nutrition Department, Desert Research Center, Cairo, Egypt
[2] Genetic Engineering and Biotechnology Research Institute, University of Sadat City, Sadate City, Menoufia, Egypt
[3] Forest Research Institute, University of Quebec in Abitibi-Temiscamingue, Rouyn-Noranda, Canada

* **Correspondence:** Email: alaa.rabee@drc.gov.eg, rabee_a_m@yahoo.com; Tel: +201096884139; Fax: +20226357858

Table S1. Physical composition (%) of the experimental rations.

Ingredients	Farm ration	Experimental rations	
	S1	S2	S3
Corn granis	22.5	10.0	10.0
Wheat grains	22.5	0	0
Soybean meal	11.0	17.0	17.0
CFM*	44	0	0
Olive cake	0	10.0	10.0
Discarded dates	0	60.0	60.0
Mineral &Vitamins	0	0.3	0.3
Salt	0	1.0	1.0
Lime stone	0	1.3	1.3
Yeast	0	0.3	0.3
Anti-fungi	0	0.1	0.1

Continued on next page

Ingredients	Farm ration	Experimental rations	
	S1	S2	S3
Total	100	100	100
Roughage	0	0	+
R: C ratio	0/100	0/100	25/75

*Note: CFM (14% CP and 65%TDN): concentrate feed mixture consisted of corn 55%, un-decorticated sunflower seed meal 12%, soybean meal 10%, wheat bran 17%, vinas 3%, lime stone 1.5%, salt 1%, premix 0.5%.

Table S2. Chemical composition of concentrate feed mixture and Date palm.

Items	Concentrate feed mixture	Date palm fronds	
	CC	UC	
Dry matter; DM (g/kg)	924.1	954.5	986.7
Crude protein; CP (g/kg DM)	200.0	185.2	75.3
Crude fiber; CF (g/kg DM)	48.0	95.1	298.4
Ether extract; EE (g/kg DM)	2.43	5.53	2.16
Nitrogen free extract; NFE (g/kg DM)	678.2	568.8	491.0
Neutral detergent fiber NDF (g/kg DM)	551.0	368.8	538.9
Acid detergent fiber; ADF (g/kg DM)	85.3	184.7	359.8
Gross energy; GE (MJ/kg DM)	18.15	18.13	16.92

Table S3. The raw data of rumen pH, VFA, and ammonia as affected by diet type.

Diet	pH	Ammonia	VFA
S1	4.99	6.71	12.40
S1	5.07	6.55	13.10
S1	4.90	6.61	13.80
S2	5.18	2.96	9.10
S2	5.25	3.09	8.90
S2	5.10	2.92	8.30
S3	5.85	3.09	8.90
S3	5.73	2.86	8.75
S3	6.15	2.94	8.60

AIMS Press

© 2022 the Author(s), licensee AIMS Press. This is an open access article distributed under the terms of the Creative Commons Attribution License (http://creativecommons.org/licenses/by/4.0)
